# Case Report: A case of paraneoplastic autoimmune encephalitis with concurrent anti-GABABR and anti-SOX1 antibody positivity

**DOI:** 10.3389/fncel.2026.1816167

**Published:** 2026-07-09

**Authors:** Feng Liu, Ye-Han Jiang, Yu-Fang Hu, Jin-Guan Qin, Guang-Sheng Liu

**Affiliations:** Longhui People’s Hospital, Shaoyang, China

**Keywords:** anti-GABABR antibody, anti-SOX1 antibody, cell-based assay, limbic encephalitis, paraneoplastic autoimmune encephalitis, small cell lung cancer, tissue-based assay

## Abstract

**Background:**

Paraneoplastic autoimmune encephalitis (PAE) is most commonly associated with small cell lung cancer (SCLC). Anti–*γ*-aminobutyric acid B receptor (GABABR) antibodies underlie a treatable limbic syndrome, whereas anti–SRY-box transcription factor 1 (SOX1) antibodies serve as high-specificity onconeural biomarkers of SCLC-driven autoimmunity. The sequential emergence of these two antibodies during the longitudinal course of a single patient has rarely been documented.

**Case presentation:**

A 75-year-old man presented with episodic impaired consciousness, behavioural disturbance and rapid cognitive decline. Brain MRI showed bilateral hippocampal signal abnormalities. A cell-based assay (CBA) detected anti-GABABR antibodies at high titre in serum (1:3200) and cerebrospinal fluid (CSF; 1:1000), while a tissue-based assay (TBA) on rat brain confirmed a neuronal cell-surface staining pattern; the full paraneoplastic panel, including anti-SOX1, was negative, and initial tumour screening was unrevealing. A diagnosis of anti-GABABR encephalitis (initially regarded as non-paraneoplastic) was made and the patient improved with intravenous immunoglobulin (IVIG), high-dose corticosteroids and maintenance mycophenolate mofetil. During a refractory second relapse 5 months later, repeat CBA showed rising anti-GABA_B_R titres (serum 1:10,000; CSF 1:1000) and newly positive anti-SOX1 antibodies (serum 1:100; CSF 1:1); a previously occult right superior mediastinal mass radiologically compatible with SCLC was identified on repeat chest CT. The diagnosis was revised to anti-GABA_B_R/anti-SOX1 dual-antibody PAE (probable PNS by 2021 PNS-Care criteria). The family declined biopsy, PET/CT and antitumour therapy because of advanced age and frailty; repeated IVIG with continued immunosuppression achieved partial clinical stabilisation with persistent cognitive impairment.

**Conclusion:**

Apparently isolated anti-GABA_B_R encephalitis can evolve into dual anti-GABABR/anti-SOX1 paraneoplastic disease, with the second antibody heralding an occult SCLC. Repeating both CBA-based and intracellular antibody panels at every clinical relapse, together with repeat thoracic imaging, is essential to avoid missing an evolving paraneoplastic aetiology.

## 1 Introduction

Autoimmune encephalitis (AE) is an increasingly recognised inflammatory disorder of the central nervous system in which autoantibodies target neuronal cell-surface or intracellular antigens (Bradshaw and Linnoila, 2018; Kerstens et al., 2024). Antibodies directed against intracellular antigens are typically associated with T-cell-mediated cytotoxic mechanisms, an underlying neoplasm, and a comparatively poorer prognosis (Smith et al., 2024; Potuznik et al., 2025). Paraneoplastic autoimmune encephalitis (PAE) is the subset of AE most strongly linked to malignancy, of which small cell lung cancer (SCLC) is by far the most frequent (Lamblin et al., 2024).

Anti–*γ*-aminobutyric acid B receptor (GABABR) antibodies define a clinically distinctive form of limbic encephalitis dominated by seizures, memory deficits, and neuropsychiatric symptoms; approximately half of all cases are paraneoplastic, almost invariably in association with SCLC (Lamblin et al., 2024; Tarrés et al., 2025). Anti–SRY-box transcription factor 1 (SOX1) antibodies, by contrast, target an intracellular nuclear transcription factor and display a high specificity for SCLC, positioning them among the most informative onconeural biomarkers in current clinical practice (Sabaka et al., 2025; Izumi et al., 2025). The two antibodies thus carry complementary information: GABABR antibodies primarily account for the neurological syndrome, whereas SOX1 antibodies provide a strong indication of an underlying tumor.

The simultaneous detection of these two antibodies has been reported in isolated patients with SCLC (Tarrés et al., 2025; Gong et al., 2022), yet the temporal evolution of dual antibody positivity—particularly its emergence during disease relapse and its relationship with subsequent tumor unmasking—remains poorly characterized.

Here, we report the case of an elderly patient who was initially diagnosed with isolated anti-GABABR encephalitis without identifiable neoplasm. During a refractory second relapse, anti-SOX1 antibodies emerged in parallel with the radiographic appearance of a previously occult mediastinal mass compatible with SCLC, ultimately establishing the paraneoplastic nature of the disease. This case illustrates the dynamic immunological landscape of PAE and underscores the importance of serial antibody testing and sustained tumor surveillance in patients with apparently non-paraneoplastic anti-GABABR encephalitis.

## 2 Case presentation

### 2.1 Ethical approval and consent

This case report was reviewed and approved by the Ethics Committee of Longhui County People’s Hospital (Approval no. KS202501). Written informed consent for participation, for publication of clinical details and for the use of all identifiable images was obtained from the patient’s legally authorised family representative, in accordance with the Declaration of Helsinki.

### 2.2 Clinical presentation and initial evaluation

A 75-year-old man was admitted on 29 July 2024 with a 3-day history of episodic impairment of consciousness and abnormal mental behaviour. Episodes arose without identifiable triggers, lasted 5–10 min, resolved spontaneously and were followed by complete amnesia. Over the same period he developed insomnia, purposeless ambulation, psychomotor agitation and delirium. His past medical history included chronic obstructive pulmonary disease, essential hypertension, type 2 diabetes mellitus and a long-standing smoking history.

On examination, the patient was lethargic with blunted affect, delayed responsiveness and disorganised speech. Cognitive testing showed marked deficits in temporal and spatial orientation, memory, calculation and comprehension. Motor strength, tone and deep tendon reflexes were preserved bilaterally, with no pathological or meningeal signs.

### 2.3 Baseline investigations

Routine investigations—complete blood count, C-reactive protein, comprehensive metabolic panel, electrolytes, thyroid function tests, erythrocyte sedimentation rate and serum tumour markers—were unremarkable. Brain magnetic resonance imaging (MRI) demonstrated bilateral hippocampal T2/FLAIR signal hyperintensities suggestive of limbic involvement (Figures 1A,B). Lumbar puncture yielded a normal opening pressure (100 mmH_2_O); CSF cell count, protein, glucose and biochemistry were within normal limits.

**Figure 1:**
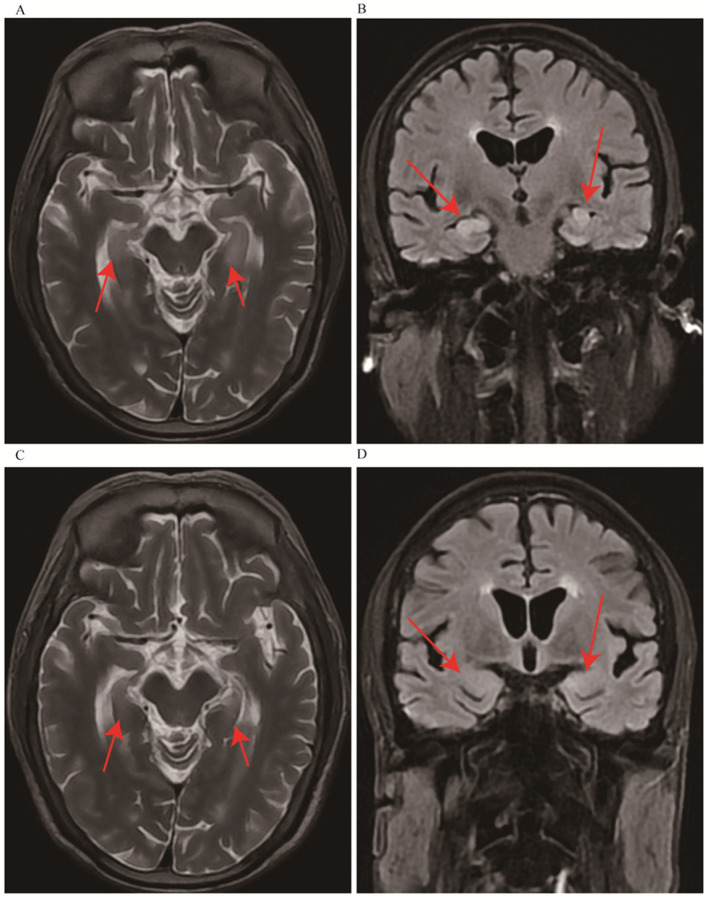
Serial brain MRI before and after first-line immunotherapy. Axial T2-weighted and FLAIR images on a 3.0-T scanner. **(A,B)** At admission (first MRI: 30 July 2024): symmetric hyperintensities confined to the bilateral hippocampi (arrows), consistent with limbic encephalitis. **(C,D)** Four weeks after IVIG and pulsed methylprednisolone (second MRI: 16 January 2025): marked attenuation of hippocampal signal without mesial-temporal atrophy, indicating a typical radiological response to first-line immunotherapy.

### 2.4 Autoantibody testing

Antibody testing was performed by Jiangsu Simcere Diagnostic Medical Co., Ltd. Neuronal cell-surface antibodies (NMDAR, AMPAR1/2, GABABR, LGI1, CASPR2, DPPX, IgLON5) were screened by a fixed cell-based assay (CBA): biochip slides spotted with HEK293 cells individually transfected with each recombinant antigen were incubated with serially diluted patient serum and undiluted-to-titred CSF, followed by fluorescein-conjugated anti-human IgG; titres were determined as the highest dilution showing specific membrane staining. Classical onconeural antibodies (Hu, Yo, Ri, CV2/CRMP5, PNMA2/Ma2, amphiphysin, recoverin, SOX1, titin, Zic4, GAD65, Tr/DNER) were initially screened by line-blot immunoassay; any positive result was retested and titrated by a confirmatory CBA. A tissue-based assay (TBA) was performed in parallel: 4-μm cryosections of perfused adult rat hippocampus and cerebellum were blocked, incubated with patient serum (starting dilution 1:200) and undiluted CSF, and visualised by indirect immunofluorescence using AlexaFluor-conjugated anti-human IgG to confirm the surface versus intracellular staining pattern and to exclude non-specific reactivity.

At baseline, CBA demonstrated anti-GABABR IgG at 1:3200 in serum and 1:1000 in CSF; all other surface and intracellular antibodies, including anti-SOX1, were negative. TBA showed a neuropil staining pattern in both serum and CSF consistent with cell-surface reactivity. Comprehensive tumour screening with chest computed tomography (CT), abdominal ultrasonography and colour-Doppler ultrasonography of superficial lymph nodes failed to identify any malignancy. The patient was therefore diagnosed with anti-GABA_B_R autoimmune encephalitis, considered non-paraneoplastic at that time.

### 2.5 Initial treatment and response

First-line immunotherapy comprised IVIG 0.4 g/kg/day for 5 consecutive days together with intravenous methylprednisolone (1,000 mg/day for 3 days, then 500 mg/day for 3 days, then 250 mg/day for 3 days), transitioned to oral prednisone 1 mg/kg/day with a 5-mg reduction every 2 weeks. Neuropsychiatric symptoms were managed symptomatically. The patient improved substantially and was discharged with normal consciousness, fluent speech, intact orientation and calculation, and only mildly impaired comprehension.

### 2.6 Relapses and emergence of dual antibody positivity

During outpatient follow-up the patient experienced two clinical relapses. Both broadly reproduced the neuropsychiatric and cognitive features observed at onset, but with additional findings on closer examination.

At the first relapse, he developed intermittent psychomotor agitation and restlessness that were markedly worse at night, together with a reduced content of consciousness, an inability to formulate answers, disorganised thinking, incoherent and disorganised speech, and a loss of effective verbal communication. This episode responded to the addition of mycophenolate mofetil 0.5 g every 12 h and maintenance oral methylprednisolone 5 mg daily.

The second relapse, occurring approximately 5 months after the initial admission, was refractory and more severe. The patient was somnolent, with persistent nocturnal agitation, restlessness and difficulty initiating sleep; he was unresponsive to questioning and showed intermittent soliloquy, disorganised thinking, incoherent speech and an inability to establish meaningful communication. In contrast to the initial presentation — at which muscle strength, tone and reflexes had all been normal — neurological examination now revealed increased muscle tone with rigidity in all four limbs, indicating evolution beyond the original limbic phenotype. Repeat lumbar puncture demonstrated an opening pressure of 150 mmH₂O — higher than the 100 mmH₂O recorded at the initial puncture but still within the normal range — with otherwise normal CSF parameters. Repeat MRI showed multiple lacunar and ischaemic lesions, diffuse white-matter degeneration and age-appropriate cerebral atrophy, without new inflammatory signal change.

Repeat antibody testing using the CBA and TBA protocols described above revealed a marked rise in anti-GABABR titres (serum 1:10,000; CSF 1:1000) and, importantly, newly positive anti-SOX1 IgG (serum 1:100; CSF 1:1) in both compartments (Figure 2). Parallel TBA again demonstrated characteristic neuropil staining (Figure 3). Prompted by these findings, a repeat chest CT identified a previously undetected right superior mediastinal mass measuring 51 × 63 mm, accompanied by multiple enlarged mediastinal lymph nodes, radiologically compatible with SCLC (Figure 4).

**Figure 2:**
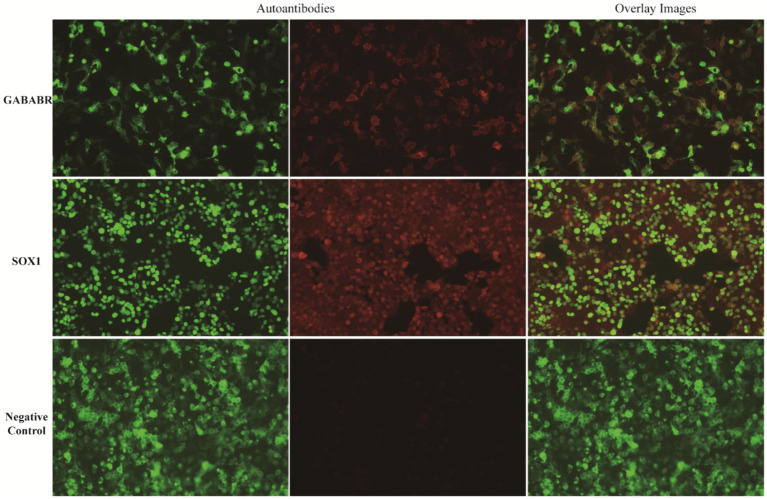
Sequential emergence of anti-GABABR and anti-SOX1 antibodies in serum by cell-based assay (CBA). HEK293 cells transfected with GABABR (B1/B2) or full-length SOX1 were incubated with serially diluted patient serum and detected with FITC-conjugated anti-human IgG; untransfected cells served as internal controls. **(A)** First admission: membrane staining of GABABR-transfected cells to 1:3200; SOX1 negative. **(B)** Second relapse: anti-GABABR titre rose to 1:10,000, with newly positive nuclear SOX1 staining at 1:100. Scale bar = 50 μm.

**Figure 3:**
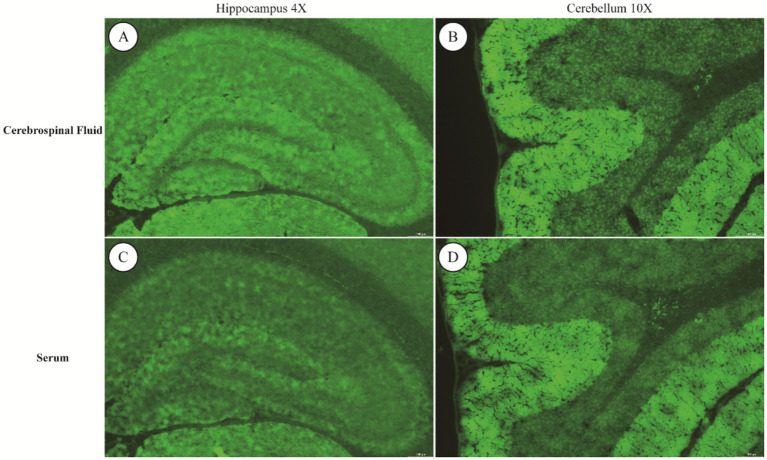
Immunoreactivity of patient serum and CSF on rat brain by tissue-based assay (TBA). Cryosections of rat hippocampus and cerebellum incubated with patient serum (1:200) or undiluted CSF, visualised by AlexaFluor-488 anti-human IgG (green) with DAPI counterstain (blue). Serum **(A,B)** and CSF **(C,D)** produced concordant neuropil staining of the hippocampal and cerebellar molecular layers with sparing of neuronal soma—a pattern typical of cell-surface antibodies and consistent with the CBA-confirmed anti-GABABR reactivity; the matching CSF pattern indicates intrathecal antibody synthesis. Scale bars = 100 μm.

**Figure 4:**
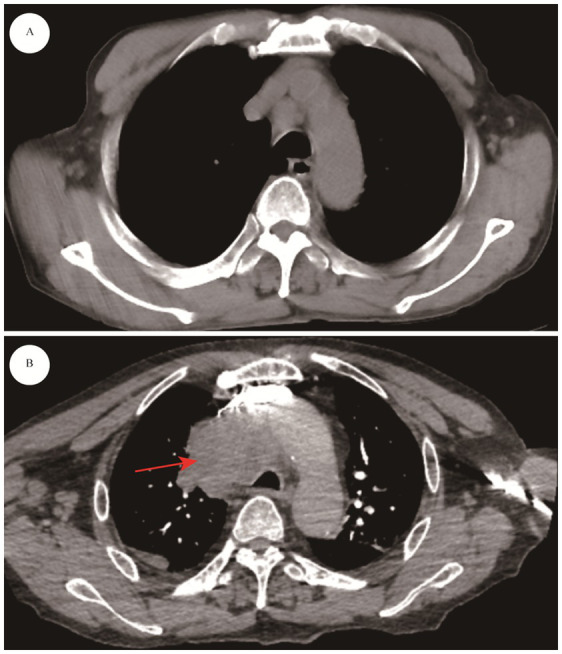
Chest CT at the first hospitalisation and second relapse.**(A)** No mass was found on the patient’s contrast-enhanced chest CT at the first hospitalisation, **(B)** A lobulated 51 × 63 mm right superior mediastinal mass (arrows) with conglomerate paratracheal and subcarinal lymphadenopathy (arrowheads) is shown—radiologically compatible with small cell lung cancer. The same region had been unremarkable on chest CT 5½ months earlier.

### 2.7 Multidisciplinary evaluation and revised diagnosis

A multidisciplinary discussion involving thoracic surgery, oncology, respiratory medicine and radiology concluded that an occult SCLC was highly probable and recommended ^18^F-FDG positron emission tomography/computed tomography (PET/CT) and tissue biopsy. Owing to the patient’s advanced age, frailty and comorbidities, the family declined biopsy, PET/CT and any antineoplastic therapy. Applying the 2021 PNS-Care diagnostic criteria, the case fulfilled the threshold for probable paraneoplastic neurological syndrome (high-risk antibody [SOX1] + intermediate-risk phenotype + radiologically identified, antibody-consistent tumour). The diagnosis was revised to PAE with concurrent anti-GABABR and anti-SOX1 antibodies.

### 2.8 Subsequent course and outcome

A repeat course of IVIG (0.4 g/kg/day for 5 days) was administered, with continued mycophenolate mofetil (0.5 g every 12 h) and low-dose oral methylprednisolone (5 mg daily). Consciousness normalised and no further episodes of impaired awareness occurred, although residual cognitive slowing persisted. Figures 1C, D show obvious resolution of bilateral hippocampal hyperintensities on follow-up scans, demonstrating a favorable radiological response to IVIG and methylprednisolone treatment. Serial outcome measures using the Clinical Assessment Scale for Autoimmune Encephalitis (CASE), the modified Rankin Scale (mRS), the Montreal Cognitive Assessment (MoCA) and the PNS-Care score are summarised in Table 1; the complete hospitalisation and treatment course is illustrated in Figure 5. Follow-up is ongoing.

**Table 1:** Serial assessments with CASE, mRS, MoCA and PNS-care scales.

Clinical time point	CASE scale score	mRS score	MoCA score	PNS-Care score
Admission (July 16th)	5	2	19	4
1 week after treatment (July 26th)	2	1	23	4
At discharge (July 30th)	1	1	23	4
3 months after discharge (October 26th)	6	4	< 18 points (Altered mental status, unable to cooperate)	4
5 months after discharge (December 30th)	20	5	< 18 points (Altered mental status, unable to cooperate)	10

**Figure 5:**
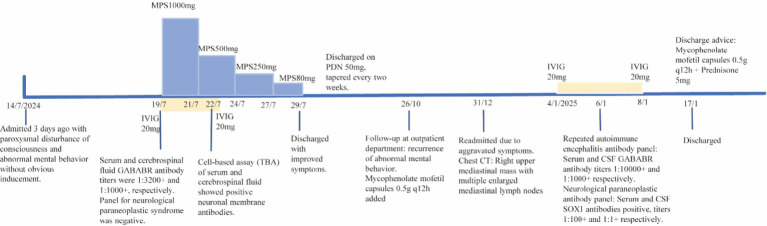
Timeline of hospitalisation, immunotherapy and antibody dynamics.

## 3 Discussion

PAE is a treatable but heterogeneous group of immune-mediated central nervous system disorders that increasingly attract clinical attention as antibody panels expand (de Bruijn et al., 2025). Single-antibody syndromes, most often anti-GABA receptor (anti-GABABR) limbic encephalitis associated with SCLC, or anti-SOX1-associated Lambert-Eaton myasthenic syndrome, paraneoplastic cerebellar degeneration or limbic encephalitis with SCLC are well characterised in the literature (de Bruijn et al., 2025). The simultaneous coexistence of a neuronal surface antibody (anti-GABABR) and a classical paraneoplastic intracellular antibody (anti-SOX1) is, by contrast, distinctly uncommon: in the literature this specific antibody pair has been documented in only a small number of patients with concurrent SCLC (Gong et al., 2022; Li et al., 2024).

Our patient is, to our knowledge, the first published case in which: (i) anti-GABABR antibodies dominated at clinical onset and the full paraneoplastic panel including anti-SOX1 was negative in both serum and CSF; (ii) anti-SOX1 antibodies emerged sequentially during a refractory second relapse, in parallel with a more than threefold rise in the serum anti-GABAₒBR titer (from 1:3200 to 1:10000+); and (iii) the radiographic appearance of an SCLC-compatible right superior mediastinal mass became visible only after this serological evolution, when serial chest CT had been repeatedly negative for 5½ months. By contrast, in the case reported by Gong et al. (2022) the anti-GABAₒBR antibody was already positive in the serum at first admission together with a PET/CT-detectable paraesophageal lesion, and anti-SOX1 antibodies became positive at the second admission 4 months later, with histological confirmation of SCLC; in the case reported by Li et al. (2024), four antibodies (anti-GABABR, anti-GAD65, anti-SOX1, anti-Ma2) were already simultaneously positive at the time of first comprehensive testing, with chest CT already showing right central lung cancer. A comparative summary of these and related dual-positive cases is provided in Table 2.

**Table 2:** Comparing the present case with all previously reported anti-GABABR + anti-SOX1 dual-positive cases.

Reference (year)	Patient (age, sex)	Presentation	Tumor (detection)	Antibodies (serum/CSF, titers, timing)	Treatment	Outcome
Gong et al. (2022), Front Immunol 2022	56 M	Seizures, rapid cognitive/memory decline	SCLC (paraesophageal mass); PET scan hypermetabolism; confirmed by biopsy	Anti-GABA 1:100 (serum, at first visit); Anti-SOX1 initially negative; 4 mo later both antibodies positive in serum and CSF: Anti-GABABR 1:100 (serum), Anti-GABABR 1:1000 (CSF) Anti-SOX1 30 AU (serum), Anti-SOX1 8 AU (CSF)	IVIG, high-dose steroids, AEDs (initial and after relapse)	Seizures controlled; cognitive symptoms improved
Li et al. (2024), BMC Neurol 2024	45 M	Dry cough, loss of consciousness, seizures, psychobehavioral abnormalities, cognitive impairment	SCLC (suspicious lung nodule on CT; biopsy with immunohistochemistry)	Serum/CSF: Anti-GABA, GAD65, SOX1, Ma2 (all positive simultaneously)	IVIG, steroids, tumor chemotherapy	Memory & mental abnormalities persisted unchanged
This report	79 M	Febrile encephalitic prodrome; confusion, cognitive decline; seizures	Small-cell lung cancer (found on imaging/biopsy)	Serum/CSF: initially anti-GABABR positive; later anti-GABABR and anti-SOX1 became positive	IVIG, IV steroids, mycophenolate mofetil therapy	Seizures and confusion improved

GABA receptors are heterodimeric G protein-coupled metabotropic receptors composed of B1 and B2 subunits, densely expressed in the hippocampus, thalamus and cerebellum, where they mediate slow inhibitory synaptic transmission. Patient-derived anti-GABABR IgG predominantly recognises the extracellular domain of the B1 subunit; in passive transfer and *in vitro* studies the antibody crosslinks and internalises the receptor and behaves as a functional antagonist, thereby reducing inhibitory tone and lowering the seizure threshold (de Bruijn et al., 2025; Lancaster et al., 2010; Dalmau et al., 2017). The clinical correlate is the limbic syndrome, prominent seizures, working-memory deficits and neuropsychiatric symptoms that our patient displayed at onset. Crucially, because the underlying lesion is largely a reversible synaptic dysfunction rather than overt neuronal death, anti-GABABR limbic encephalitis is typically responsive to first-line immunotherapy, as it was in our patient (full recovery of MoCA from 19/30 to 23/30 after intravenous immunoglobulin and methylprednisolone). Approximately 50% of anti-GABABR cases are paraneoplastic, with SCLC being by far the most common associated tumour (Höftberger et al., 2013; Zhu et al., 2020; McKay et al., 2019).

SOX1 is an intracellular SRY-box transcription factor selectively expressed in Bergmann glia of the cerebellar Purkinje cell layer and ectopically expressed by SCLC. Because the antigen is intracellular, anti-SOX1 IgG cannot reach its target in intact neurons and is widely regarded as non-pathogenic; rather, its detection serves as a high-specificity biomarker of an underlying SCLC-driven antitumour immune response (Sabater et al., 2008; Sun et al., 2020; Arnaldos-Pérez et al., 2023). The neurological damage in anti-SOX1-positive patients is therefore thought to be mediated mainly by CD8^+^ cytotoxic T cells that recognise SOX1-derived peptides cross-presented in both tumour and CNS, producing perivascular and parenchymal T cell infiltrates and cumulative, partly irreversible neuronal loss (Bien et al., 2012; Masciocchi et al., 2024; Greenlee et al., 2021). The detection of anti-SOX1 antibodies in our patient at the second relapse therefore signalled, in retrospect, both an occult SCLC-compatible tumour and an additional, T cell-driven component of CNS injury — consistent with the patient’s incomplete recovery of cognitive function despite immunotherapy.

Why two antibodies arise sequentially in the same patient is best explained by the well-described phenomenon of intermolecular epitope spreading. The current model posits that tumour cells ectopically express CNS antigens; antigen-specific dendritic cells, T cells and B cells converge in tumour-draining lymph nodes, where germinal-centre reactions generate affinity-matured plasma cells (de Bruijn et al., 2025; Dalmau et al., 2017; Masciocchi et al., 2024). Ongoing antibody or T cell-mediated injury to neuronal tissue subsequently releases additional intracellular antigens (including SOX1) into the immune compartment, where they can be presented to new T cell and B cell clones, a process strongly supported by HLA class II-mediated CD4^+^ T cell help. The temporal pattern in our patient is consistent with this sequence: the surface-targeted anti-GABABR response was established first and produced an initially reversible limbic syndrome; persistent antitumour pressure, possibly amplified by suboptimal immunosuppression because of the patient’s frailty, then drove the emergence of the intracellular anti-SOX1 response, which became detectable only when its titer reached commercial assay thresholds (serum 1:100; CSF 1:1).

The two antibodies are therefore best regarded as acting through parallel, biologically distinct pathways. Anti-GABABR antibodies drive the reversible, seizure-dominant component of the syndrome; the anti-SOX1 response indexes an active, predominantly T cell-mediated antitumour reaction whose neuronal-collateral component is largely irreversible. This dual-pathway interpretation predicts a partial response to immunotherapy with stepwise cognitive decline at each relapse and is consistent with the observation that multi-antibody-positive paraneoplastic AE generally has a poorer prognosis than single-antibody disease (Höftberger et al., 2013; Qiao et al., 2022; Hardy-Werbin et al., 2018). It also provides a mechanistic rationale for combining anti-tumour therapy (when feasible) with immunotherapy that can target both arms of the response.

Our case carries three diagnostic lessons. First, a positive neuronal surface antibody does not exclude a paraneoplastic process; conversely, a positive classical paraneoplastic antibody does not exclude a co-existing surface antibody. Comprehensive panels covering both surface and intracellular targets should therefore be considered whenever the clinical context raises the possibility of a paraneoplastic process — for example in older smokers, in patients with an atypical or treatment-refractory course, or when the phenotype and the antibody result are not fully concordant. Conversely, in patients with a typical clinical phenotype, a compatible positive antibody and a good response to first-line immunotherapy, testing for the full complement of both surface and intracellular antibodies may not be mandatory, and the breadth of the panel should ultimately remain a matter of clinical judgement (Masciocchi et al., 2024; Budhram et al., 2020; Dalmau and Graus, 2023). Second, antibody profiles can evolve. Repeating the panel at every clinical relapse — or at any unexplained change in disease course — is essential and may unmask a previously occult paraneoplastic etiology, as it did in our patient. Third, an initially negative tumour screen does not rule out malignancy: small mediastinal SCLC and small ovarian teratomas in particular can lie below the detection threshold of conventional CT and abdominal ultrasound for months (de Bruijn et al., 2025; Titulaer et al., 2011). Where antibody profile and clinical phenotype point strongly to a paraneoplastic syndrome, repeat imaging including 18F-FDG PET/CT should be performed. Applying the updated PNS-Care criteria (Graus et al., 2021), our patient achieves “probable” paraneoplastic neurological syndrome status (high-risk antibody [SOX1] + intermediate-risk phenotype + radiographically identified, antibody-consistent tumour), notwithstanding the absence of histological confirmation.

The therapeutic course of our patient reinforces several principles. (i) First-line immunotherapy (IVIG + high-dose corticosteroids) produced rapid and substantial improvement, as expected for a surface-antibody-dominant phase. (ii) Maintenance with low-dose corticosteroids plus mycophenolate mofetil prevented seizure recurrence but did not prevent emergence of the intracellular SOX1 response, in keeping with the limited efficacy of B cell-sparing immunosuppression against ongoing tumour-driven T cell-mediated autoimmunity. (iii) The dramatic rise in anti-GABABR titer at the second relapse, coincident with anti-SOX1 seroconversion and radiographic tumour appearance, illustrates that without tumour-directed therapy the autoimmune stimulus remains unabated — a pattern repeatedly emphasised in larger paraneoplastic cohorts (de Bruijn et al., 2025; Sun et al., 2020). (iv) In frail patients in whom chemotherapy is not feasible, sustained immunotherapy and meticulous symptomatic management may still achieve clinically meaningful stabilisation, as in our case, even though the long-term prognosis remains guarded.

Referring to EFNS guidelines (Titulaer et al., 2011), updated PNS-Care criteria (Graus et al., 2021) and a recent review (de Bruijn et al., 2025), we establish a surveillance schedule for patients with dual anti-GABABR and anti-SOX1 positivity: contrast-enhanced thoraco-abdominopelvic CT is performed every 3 months in the first year, every 6 months in years 2–3 and annually for no less than 5 years in total; whole-body ^18^F-FDG PET/CT is conducted at baseline, on clinical relapse, or when antibody levels persist or rise with negative CT findings. Serum and CSF antibody panels including anti-GABABR, anti-SOX1 and full PNS markers are tested at baseline and relapse, with serum titers measured every 3 months in the first year and every 6 months thereafter. MMSE, MoCA, CASE and mRS assessments are completed at 3-monthly visits, formal neuropsychological tests are arranged at 6, 12 months and yearly afterwards, while routine screening for fatigue, REM sleep behaviour disorder, depression and anxiety, common sequelae of autoimmune encephalitis, is also performed.

This case has three principal limitations. First, the suspected mediastinal SCLC was never histologically confirmed because the family declined biopsy, PET/CT and antineoplastic therapy in view of the patient’s advanced age and comorbidities; the paraneoplastic etiology therefore rests on the combination of antibody profile and radiology, although it reaches “probable” status by formal criteria. Second, anti-SOX1 testing relied on a commercial CBA, and low-titer false positives are known to occur with this antibody (Masciocchi et al., 2024; Ruiz-García et al., 2019); the consistent serum-and-CSF positivity, the rising serum titer and the supporting radiology nevertheless make a true positive result highly likely. Third, follow-up so far covers approximately 12 months; longer follow-up will be required to confirm the late natural history of dual-antibody disease in patients managed without antineoplastic therapy.

## 4 Conclusion

This case illustrates that the sequential emergence of anti-SOX1 antibodies in a patient with suspected anti-GABABR limbic encephalitis can constitute the earliest accessible signal of a suspected occult SCLC-associated malignancy, even when serial conventional imaging has been negative. The two antibodies are mechanistically complementary: anti-GABABR mediates a reversible, seizure-dominant synaptic syndrome, whereas anti-SOX1 indexes an active, T cell-driven antitumour response that contributes a cumulative, partly irreversible cognitive component. Clinicians caring for older patients with autoimmune encephalitis should therefore (i) request paired serum-and-CSF surface and intracellular antibody panels at every relapse, (ii) maintain a low threshold for repeat oncological imaging including 18F-FDG PET/CT. Future studies that combine longitudinal multi-antibody profiling with cellular and humoral immunophenotyping will be needed to test the epitope-spreading model directly and to refine treatment for this rare but instructive patient population.

## Data Availability

The original contributions presented in the study are included in the article/supplementary material, further inquiries can be directed to the corresponding author.
